# Altered Innate Immunity and Damaged Epithelial Integrity in Vaginal Microbial Dysbiosis

**DOI:** 10.3389/frph.2022.876729

**Published:** 2022-07-13

**Authors:** Ryan K. Cheu, Avid Mohammadi, Luca Schifanella, Courtney Broedlow, Connor B. Driscoll, Charlene J. Miller, R. Keith Reeves, Mark H. Yudin, Tiffany Hensley-McBain, Rupert Kaul, Nichole R. Klatt

**Affiliations:** ^1^Department of Pharmaceutics, University of Washington, Seattle, WA, United States; ^2^Washington National Primate Research Center, University of Washington, Seattle, WA, United States; ^3^Department of Pediatrics, University of Miami Miller School of Medicine, Miami, FL, United States; ^4^Departments of Medicine and Immunology, University of Toronto, Toronto, ON, Canada; ^5^Division of Surgical Outcomes and Precision Medicine Research, Department of Surgery, University of Minnesota, Minneapolis, MN, United States; ^6^Center for Virology and Vaccine Research, Beth Israel Deaconess Medical Center and Harvard Medical School, Boston, MA, United States; ^7^Division of Innate and Comparative Immunology, Department of Surgery, Duke University, Durham, NC, United States; ^8^Department of Obstetrics and Gynecology, University of Toronto, Toronto, ON, Canada; ^9^Department of Medicine, University Health Network, Toronto, ON, Canada; ^10^McLaughlin Research Institute, Great Falls, MT, United States

**Keywords:** bacterial vaginosis, neutrophils, female reproductive tract, epithelial barrier damage, vaginal microbe, women health

## Abstract

The role of neutrophils relative to vaginal dysbiosis is unclear. We hypothesize that bacterial vaginosis (BV)-associated bacteria may induce the activation and accumulation of mucosal neutrophils within the female reproductive tract (FRT), resulting in epithelial barrier damage. We collected endocervical cytobrushes from women with and without BV and assessed bacteria community type and frequency/functional phenotypes of neutrophils. We performed *in vitro* whole blood co-cultures with BV-associated bacteria and healthy vaginal commensals and assessed their impact on epithelial integrity using transepithelial electrical resistance. We demonstrated increased neutrophil frequency (*p* < 0.0001), activation (*p* < 0.0001), and prolonged lifespan (*p* < 0.0001) in the cytobrushes from women with non-*Lactobacillus* dominant (nLD) communities. Our *in vitro* co-cultures confirmed these results and identified significant barrier damage in the presence of neutrophils and *G. vaginalis*. Here, we demonstrate that BV-associated bacteria induce neutrophil activation and increase lifespan, potentially causing accumulation in the FRT and epithelial barrier damage.

## Introduction

Bacterial vaginosis (BV) has been linked to an increased risk of Sexual Transmitted Infections (STIs) and Human Immunodeficiency Virus (HIV) acquisition and forward transmission and to other adverse outcomes such as preterm delivery and pelvic inflammatory disease (1–6). BV is the most common cause of vaginal discharge amongst women of reproductive age and is directly related to the make-up of the vaginal flora (7). Specifically, clinical and molecular BV are characterized by a diverse community of anaerobes, including including *Gardnerella vaginalis, Mobiluncus* spp., *Prevotella* spp., *Fannyhessea vaginae* (formerly known as *Atopobium vaginae*), and others (1, 8, 9). In contrast, *Lactobacillus* species (such as *L. crispatus, L. jensenii* and *L. gasseri*) dominate an optimal vaginal microbiome (10). Vaginal communities dominated by *Lactobacillus iners* tend to be better than the polymicrobial anaerobic bacterial communities; however, even in this context, the species of *Lactobacillus* have important clinical implications, with *L. iners* being more inflammatory and less protective against HIV acquisition than other *Lactobacillus* spp., perhaps due to differences in hydrogen peroxide production and differing capacity to protect against other bacterial and viral STIs (11). BV is commonly diagnosed clinically using Amsel's criteria (12). In Amsel's criteria, BV is diagnosed when three out of four criteria are met: abnormal discharge, pH > 4.5, clue cells present, and fish odor (12). Nugent score is another method used to diagnose BV, which attempts to capture the bacterial morphotypes *via* gram staining due to the differentiating *Lactobacillus-*dominating communities compared with small and curved Gram-variable rods (12). Nugent scoring is widely used to define BV in large cohorts. With increasing correlations to adverse health outcomes, there is a growing need for improvement in BV diagnosis. Due to advances in DNA sequencing technology, molecular methods using sequencing and quantitative PCR are becoming more readily available, as seen with an FDA-approved molecular diagnostic BV test (13). These techniques have identified even a larger proportion of asymptomatic women at risk for cervicovaginal inflammation and increased STI risk due to the ability of molecular BV testing to identify specific taxon (14).

BV has been found associated with the prevalence and incidence of multiple STIs, including chlamydia, gonorrhea, herpes, and trichomoniasis (15–19). Molecular BV is very common amongst reproductive-age women, particularly black women in both North America and Africa, with a prevalence as high as 63% reported in Zambia (20, 21). Amsel's criteria, Nugent score, and molecular techniques, while overlapping, offer different depths to capture better the cervicovaginal microbiota. With increasing associations of BV and inflammation or risk of STIs, including HIV, and the growth in molecular techniques, there is a clear need to associate better specific taxa with these adverse outcomes. BV is associated with a 60% increase in HIV incidence (8, 9, 22), but the mechanisms underpinning this association are not clearly understood.

The mucosal barrier provides protection against invading bacterial and viral pathogens, including HIV (23), and inflammation-induced reductions in epithelial barrier integrity may be an important mechanism by which BV increases susceptibility to HIV infection (23). Neutrophils are within the first responders to pathogen and play a crucial role in antibacterial and antifungal defense, but they can also contribute to barrier damage and inflammation (24). Therefore, there is a delicate balance between the anti-microbial activity of neutrophils and potential tissue damage by releasing harmful effector molecules such as reactive oxygen species (24). Furthermore, more studies have highlighted the importance of balance within neutrophil functions (24–30) and have linked increased neutrophil proteases with inflammatory cytokines and with barrier function and integrity (23, 31–34).

This phenomenon can impact HIV and STIs mucosal acquisition. There have been discrepancies in whether neutrophils increase or decrease during BV, as seen in Cauci et al., where they did not observe an increase in neutrophil numbers in women with BV (35). However, studies found increases in IL-8, a potent chemotactic and activating factor for neutrophils, in vaginal fluid from women with BV (36–38). Even when compared with candidiasis, studies have shown that women with BV have lower levels of neutrophils (39, 40). These discrepancies may be attributed to the aforementioned issues with diagnosing BV. For this reason, in our study we utilized molecular techniques to identify specific taxa and multiparameter flow cytometry to identify accurately neutrophil frequency and phenotype.

In here we analyze the effects of vaginal microbiota composition and specific BV-associated bacteria on neutrophil lifespan and functional phenotypes. Based on previous work, we used epithelial barrier assays to fully evaluate the effect of neutrophils on barrier integrity in the presence of these taxa, revealing potential mechanisms for increased HIV susceptibility amongst women with BV (41).

## Materials and Methods

### Study Procedures

Informed written consent was obtained from all participants prior to enrolment, and the study was approved by Institutional Review Boards at St. Michael's Hospital (Toronto) and the University of Toronto. The described studies were conducted according to the principles expressed in the Declaration of Helsinki. For analysis of endocervical neutrophil populations, female participants were recruited from the Colposcopy Clinic at St. Michael's Hospital, Women's Health Care Centre in Toronto, Canada (CIHR #TMI-138656). Recruited participants (*n* = 6) self-reported to be HIV-negative and were not actively menstruating at the time of sample collection; women with/without clinical findings of BV were recruited using a convenience-based cross-sectional sampling frame. For the blood samples for bacterial stimulations, HIV-negative study participants (*n* = 6) were recruited through the University of Washington Center for AIDS Research. Informed written consent was obtained from all participants prior to enrolment, and the study was approved by Institutional Review Boards at St. Michael's Hospital (Toronto) and the University of Toronto. The described studies were conducted according to the principles expressed in the Declaration of Helsinki.

### Sample Collection

Two cervical cytobrushes were collected from each participant after inserting a cytobrush into the endocervical os and rotation through 360°. Cytobrushes were then transferred into 1 mL of Roswell Park Memorial Institute (RPMI) in a 15mL conical tube and vortexed for 30 seconds prior to removing and disposing of the cytobrush. The cytobrush media was centrifuged at 1,900 RPM for 6 min at 4°C. Cytobrush supernatant was then aliquot into two cryovials (500 μL/vial) and stored immediately at −80°C. Vaginal swabs were inserted into the vaginal speculum and rotated 360° three times prior to collection. Swabs were then placed back into the original container and immediately stored at −80°C.

### Flow Cytometry

Cytobrush cell pellets were immediately re-suspended in 500 μL PBS prior to flow cytometry staining. Whole blood samples were stained fresh or following co-culture with various bacteria as outlined below. Cytobrush cells were stained using the following surface antigen mouse anti-human antibodies: CD32 FITC (Becton Dickinson, BD), CD66b PerCP-Cy 5.5 (BD), CD64 Ax700 (BD), CD11b APC Cy7 (BD), CD3 PE (BD), CD45 PE CF594 (BD), Caspase-3 V450 (BD), CD16 BV605 (BD), CD15 BV650 (BD), HLA-DR BV711 (BD), CD14 BV786 (BD), CD20 BUV395 (BD), CD49d BV421 (BD), CD89 APC (Biolegend), CD62L PerCP Efluor 710 (eBioscience), CD 274 (PD-1L) PE Cy7 (Biolegend), and Aqua L/D/eBio506 L/D (LifeTech). Blood from the bacteria stimulations were stained using the following Becton Dickinson (BD), Biolegend, eBioscience, and LifeTech surface antigen mouse anti-human antibodies: CD32 FITC (BD), CD45 PerCP (Biolegend), CD11b APC Cy7 (BD), CD3 PE (BD), CD45 PE CF594 (BD), CD62L PerCP Efluor 710 (eBioscience), CD 274 (PD-1L) PE Cy7 (Biolegend), Caspase-3 V450 (BD), CD20 BV570 (Biolegend), CD16 BV605 (BD), CD15 BV650 (BD), HLA-DR BV711 (BD), CD14 BV786 (BD), and Aqua L/D/eBio506 L/D (LifeTech). Both cells from cytobrushes and blood were permeabilized using Cytofix/Cytoperm (BD) after surface staining. After staining, samples were fixed in 1% paraformaldehyde and collected with the following two flow cytometers: Fortessa X20 (BD) and an LSR II (BD) for the cytobrush samples and *in vitro* stimulations, respectively. FlowJo version 9.7.6 was used for analysis.

### Bacterial Strains and Culture Conditions

All bacterial strains used in the study belonged to American Type Culture Collection (ATCC) and the stated the ATCC strains. *Lactobacillus iners* ATCC 55195, *Lactobacillus crispatus* ATCC 33197, *Gardnerella vaginalis* ATCC 14018 (group C), *Prevotella bivia* ATCC 29303, and *Fannyhessea vaginae* (previously known as *Atopobium vaginae*) ATCC BAA-55, were obtained from the American Type Culture Collection (ATCC). *Lactobacillus* spp. and *G. vaginalis* were maintained on Human Bilayer Tween Agar (BD) plates and New York City III (NYCIII) medium according to the manufacturer's instructions. *P. bivia* and *A. vaginae* were maintained on ATCC medium 260: Trypticase soy agar/broth with defibrinated sheep blood and ATCC medium 1377: Haemophilus ducreyi medium, respectively. Agar plates and liquid cultures were incubated at 37°C with anaerobic gas mixture, 80% N_2_, 10% CO_2_, and 10% H_2_. Frozen stocks of strains were stored at −80°C in 40% (v/v) glycerol.

### *In vitro* Bacterial Stimulations

For the *in vitro* stimulations, 100 μL of whole blood from healthy individuals was stimulated at a ratio of 2.5 bacteria per leukocyte in 1mL R10 media (RPMI 1640 with 2.05 mM L-glutamate and 10% fetal bovine serum). Incubations were done at 37°C for 18 h. Following incubation, blood was centrifuged and washed with 1 mL PBS prior to flow cytometry analysis, as described above.

### TEER w/Neutrophil Isolation

HeLa cells (ATCC CCL-2) were cultivated and seeded into transwell inserts (Corning). Cells were monitored microscopically to evaluate detachment. Wells were seeded with 50,000 HeLa cells in 300 μL of Dulbecco's Modified Eagle's Medium (DMEM, ATCC 30-2002) and 10% fetal bovine serum. The wells were filled with 1 mL of the same medium and placed at 37°C until stable TEER values (40–60 Ω.cm^2^), and a monolayer was formed.

Triplicate technical replicates were taken for each well. Nine biological replicates were assessed per condition. Cutoff values were <400 Ω. Resistance was measured using an EVOM2 Epithelial Voltohmeter (World Precision Instruments). Neutrophils were isolated from whole blood using the MACSxpress Whole Blood Neutrophil Isolation Kit (Miltenyi Biotec) and Red Blood Cell Lysis solution. Neutrophil purity was validated after staining an aliquot of sample fraction with anti-CD15 and assessed *via* flow cytometry. Neutrophils were then counted and 2 × 10^5^ were added to the wells. Bacteria were added at a 2:1 ratio with neutrophils and the wells were placed at 37°C. TEER readings were done at 0, 4, 18, and 24 h.

### Lucifer Yellow Transport Assay

Transport studies were performed on HeLa cell monolayers with 100 μg/mL lucifer yellow (Thermo Fisher) (LY). LY is a marker used to determine the apparent paracellular permeability. As LY is a small, hydrophilic molecule, it can easily diffuse through passive paracellular diffusion. In this case, we use LY has a confirmation of tight junction formation in a monolayer, a vital control for transwell assays. An experiment without a sample added to the apical side was performed as a negative control. Fresh DMEM media was added to both sides of the transwell. After a 15-min rest period, 0.5 mL DMEM media containing LY was added to the apical chamber, while 1.5 mL of DMEM media without LY was added to the basolateral side. After incubation for 1 h, transport rates were determined by measuring fluorescence from LY on the basolateral side. The LY was determined fluorometrically at 430 nm excitation and 540 nm emission using a SpectraMax Gemini XS (Molecular Devices). HeLa cell tight junction formation was confirmed using LY fluorometric data. HeLa cells had an average permeability of 0.010 ± 0.001 nmol/min/cm^2^ while media alone had an average permeability of 0.062 ± 0.011 nmol/min/cm^2^.

### Analysis of 16s rRNA Gene Sequencing Data

Vaginal swabs were added to 200 μL of the Qiagen's DNeasy Blood and Tissue kit lysis buffer (ATL). Samples were heated at 65°C for 10 min before lysozyme solution was added to a final concentration of 10 mg/ml. A 1-h incubation at 37°C then occurred, followed by adding 5% SDS to a final concentration of 1% w/v. 10-min incubation at 56°C was then done. Twenty-five microliter of Proteinase and 200 μL of Buffer AL from the Qiagen's DNeasy Blood and Tissue kit was then added, and incubation of 30 min at 56°C took place. The swabs were removed, and 200 μL of EtOH was added to the solution. The rest of the DNA extraction was performed according to Qiagen's DNeasy Blood and Tissue Kit protocol. The Earth Microbiome Protocol for 16S Illumina sequencing with primers discussed in Caporaso et al. (42) was used to amplify the V4 region of the 16S SSU rRNA. Amplicon concentrations were normalized, pooled, and cleaned, followed by a KAPA quantification. A 2 × 150 bp Illumina MiSeq run was used to sequence the pooled library. 16S sequencing reads were processed using QIIME2 version 2018.2; taxonomic determination in QIIME2 utilized the Silva 119 classifier. Taxonomic plots were created in part within Rstudio utilizing the phyloseq package (43).

### Statistical Analysis

GraphPad Prism statistical software (version 6; GraphPad Software, San Diego, CA) was used for all statistical analyses. Differences in active Caspase-3, neutrophil frequencies, and CD62L between *Lactobacillus* dominant and non-*Lactobacillus* dominant communities from the cervicovaginal cytobrushes were determined by Mann-Whitney *U-*test. A one-way ANOVA was used to assess differences between groups stimulated with different bacteria and controls followed by a Tukey's multiple comparisons. A one-way ANOVA was also used to assess differences between groups stimulated with different bacteria and neutrophils for the TEER assays followed by a Tukey's multiple comparisons. Relative abundance plots focused on the top 21 most abundant genera.

## Results

### Microbial Composition of BV Positive and BV Negative Women

Nugent score is effective in diagnosing BV by assessing bacterial morphology using gram staining through the absence of *Lactobacillus*-dominant rods. However, with some BV-associated bacteria resulting in gram-variable staining, it is difficult to identify specific non-*Lactobacillus* taxon (12). Similarly, the scoring system allows for intermediate diagnosing where combinatory morphology types exist. 16S sequencing allows for the complete characterization of the microbiota using deep sequencing of the 16s rRNA gene and, in doing so, allows the measurements of relative abundance of bacterial taxa.

**Figure 1:**
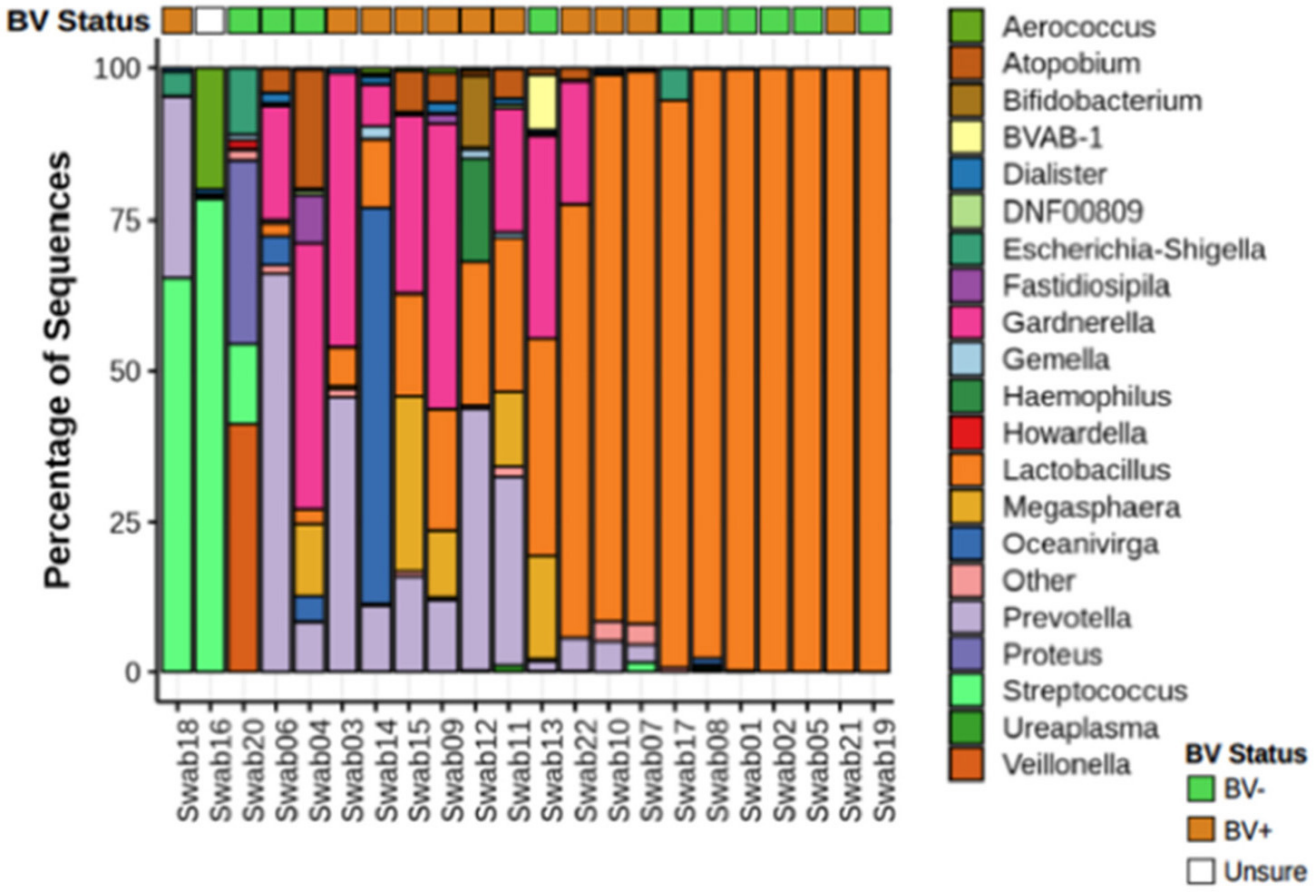
Microbial composition from vaginal swabs. Relative abundance of bacteria from vaginal swabs from women with and without diagnosed BV. Nugent score intermediate was not take in consideration. The 21 most abundant genera are shown. *Atopobium* genera have now been changed to *Fannyhessea*. Name was left as *Atopobium* in the figure to keep consistent with 16S database used for taxonomic assignment.

We analyzed vaginal swabs from 22 women using 16S ribosomal RNA (rRNA) sequencing. This analysis identified 229 different bacterial genera. Ten women were diagnosed as BV negative, with a Nugent score under seven, and 11 women were diagnosed as BV positive based on a Nugent score above seven. One woman's diagnosis was inconclusive by Nugent scoring. All these women were healthy, HIV negative individuals. Two major bacterial community groups were identified: one in which *Lactobacillus* represented >50% of the total bacterial composition (*Lactobacillus* dominant, LD, *n* = 10 women), and the other dominated by non-*Lactobacillus* microbiota (nLD, *n* = 12 women) (Figure 1).

### Effect of Vaginal Dysbiosis on Neutrophil Lifespan and Accumulation

**Figure 2:**
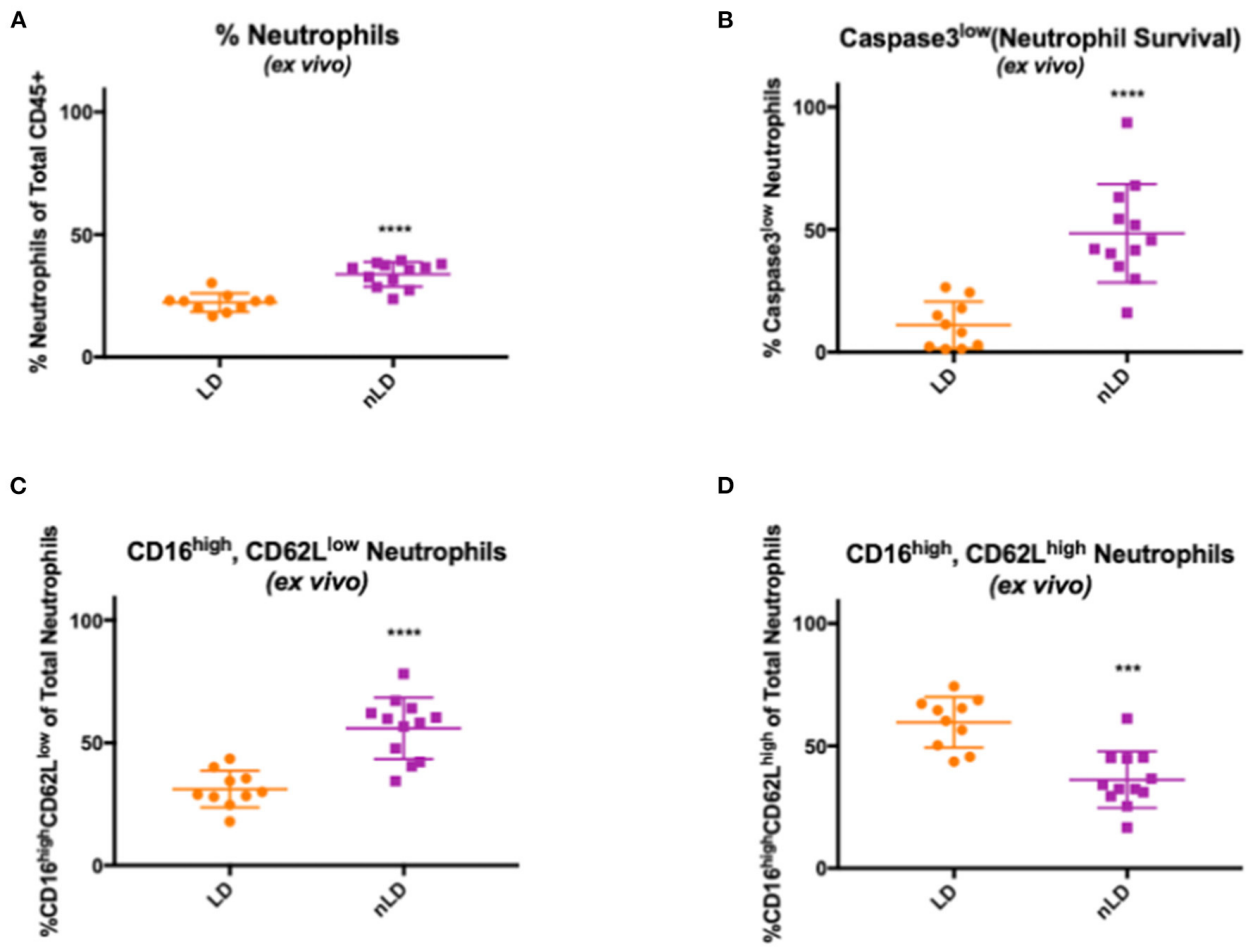
Effects of vaginal bacteria from cervico-vaginal cytobrushes on neutrophil frequency and phenotype. Neutrophils frequency in cervico-vaginal cytobrushes obtained from 22 women divided in two groups according to the vaginal bacterial composition: *Lactobacillus* dominant (LD, *n* = 10 women) and non-*Lactobacillus* dominant (nLD, *n* = 12 women) **(A)** Higher frequency of total neutrophils in nLD compared to LD as a percentage of total CD45+ leukocytes in vaginal cytobrushes measured by flow cytometry (*p* < 0.0001). **(B)** Higher frequency of active Caspase-3low neutrophils in nLD compared to LD as a percentage of total neutrophils in vaginal cytobrushes measured by flow cytometry (*p* < 0.0001). **(C)** CD16high, CD62Llow neutrophils in nLD compared to LD as a percentage of total neutrophils in vaginal cytobrushes measured by flow cytometry (*p* < 0.0001). **(D)** Lower frequency of CD16high, CD62Lhigh neutrophils in nLD compared to LD as a percentage of total neutrophils in vaginal cytobrushes measured by flow cytometry (*p* = 0.0002). In **(A–C)** “****” represents *p*-values < 0.0001, in **(D)** “***” represents *p*-value = 0.0002. Statistical differences in neutrophil frequencies between nLD and LD samples were determined by Mann-Whitney test with a *p*-value < 0.05 considered significant. Lines represent mean and SD.

To identify the effects of vaginal microbial communities on neutrophil frequencies and lifespan, we further analyzed cervicovaginal cytobrushes using a multicolor flow cytometry-based approach to assess the frequency of neutrophils as a percentage of live CD45+ leukocytes. In our nLD samples, we observed a significantly higher frequency (*p* < 0.0001) of total neutrophils amongst live CD45+ cells compared to our LD samples (Figure 2A). To assess increased neutrophil lifespan as a potential mechanism for this accumulation, we evaluated levels of active Caspase-3 and levels of CD16 in neutrophils to determine the frequency of surviving, functional neutrophils (CD16high, Active Caspase-3Low). In our nLD samples, we observed a significant increase in the number of neutrophils that had downregulated both CD16 and Caspase 3 (*p* < 0.0001) compared to LD samples (Figure 2B), indicating that they were not in apoptosis but would survive, likely the mechanism underlying accumulation in the FGT. To further identify the effects of vaginal dysbiosis on neutrophils, leukocytes from cervicovaginal cytobrushes were analyzed using a multicolor flow cytometry staining panel designed to assess specifically neutrophil phenotype. We identified neutrophils previously reported as having suppressive function (44) by assessing the frequency of CD62LlowCD16High neutrophils. In our non-*Lactobacillus* (nLD) samples, we observed a significant increase (*p* < 0.0001) in these potentially suppressive neutrophils compared to samples from women with a *Lactobacillus* dominant (LD) community (Figure 2C). As such, we also observed significantly more (*p* = 0.0002) CD62LhighCD16high neutrophils, representing a less activated neutrophil population, in the LD samples when compared to the nLD samples (Figure 2D).

### Bacteria Associated With Vaginal Dysbiosis Impact Neutrophil Lifespan and Accumulation

**Figure 3:**
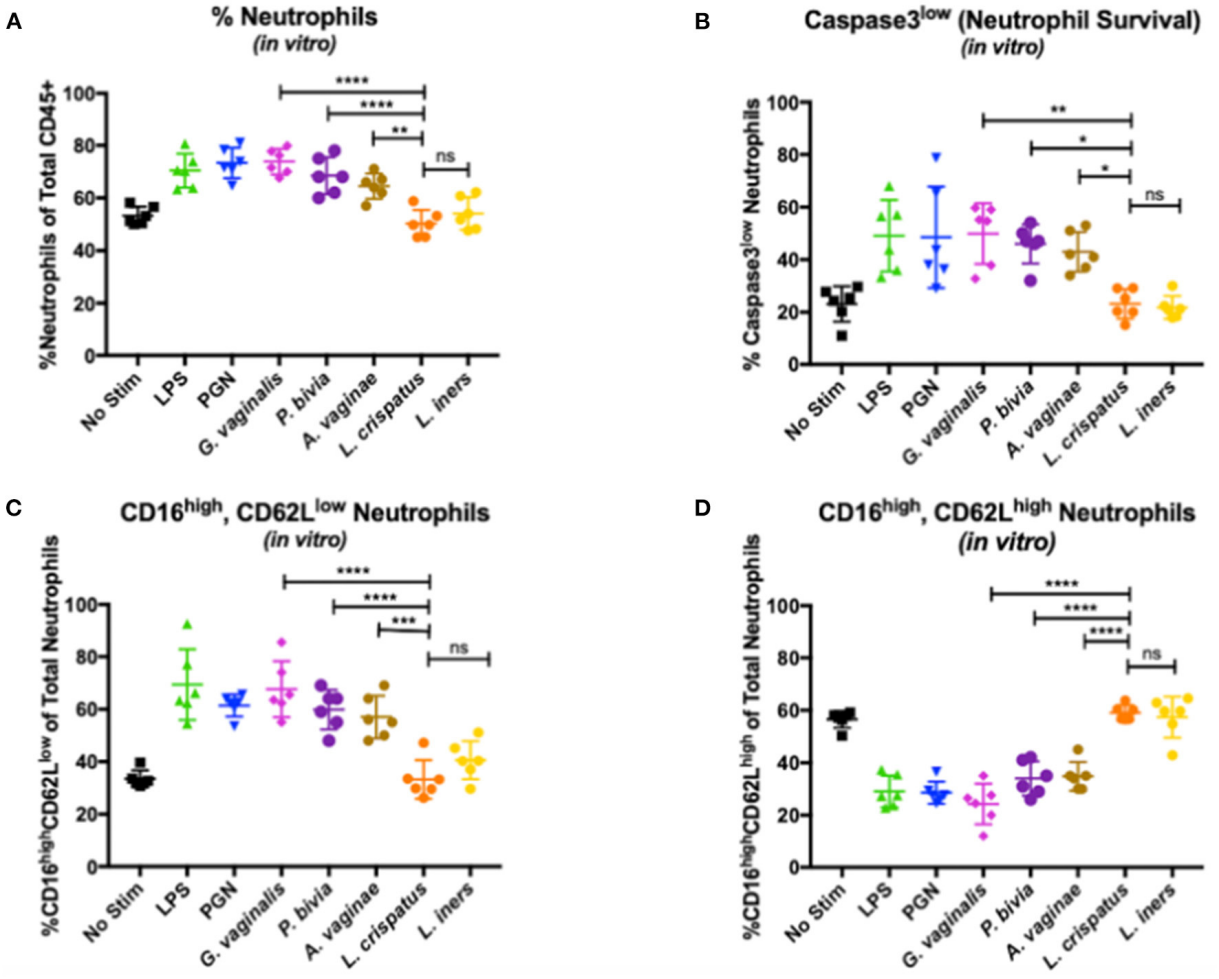
Effects of vaginal bacteria on neutrophil frequency and phenotype. **(A)** Neutrophils as a percentage of total CD45+ leukocytes measured by flow cytometry. Higher frequency of neutrophils in co-cultures with *G. vaginalis, P. bivia*, and *A. vaginae* compared to samples cultured with *L. crispatus* (respectively *p* < 0.0001, represented as **** in the figure; *p* < 0.0001, represented as **** in the figure; and *p* = 0.0018, represented as ** in the figure). No significant difference between samples co-cultured with *L. iners* and *L. crispatus* (*p* = 0.9321 represented as ns in the figure). **(B)** Active Caspase-3low as a percentage of total neutrophils measured by flow cytometry. Higher percentage of non-apoptotic neutrophils in samples co-cultured with *G. vaginalis* compared to samples cultured with *L. crispatus* (*p* = 0.0021, represented as ** in the figure), *P. bivia* compared to samples cultured with *L. crispatus* (*p* = 0.0126, represented as * in the figure) and *A. vaginae* compared to samples cultured with *L. crispatus* (*p* = 0.0452, represented as * in the figure). No significant difference between samples cultured with *L. crispatus* and *L. iners* (*p* > 0.9999, represented as ns in the figure) **(C)** CD16high, CD62Llow neutrophils as a percentage of total neutrophils measured by flow cytometry. Increase in CD16highCD62Llow neutrophils in samples co-cultured with *G.vaginalis* compared to samples cultured with *L. crispatus* (*p* < 0.0001, represented as **** in figure), in samples co-cultured with *P. bivia* compared to samples cultured with *L. crispatus* (*p* < 0.0001 represented as **** in figure) and in samples co-cultured with *A. vaginae* compared to samples cultured with *L. crispatus* (*p* = 0.0004, represented as *** in figure). No significant difference between the co-cultures with *L. crispatus* and *L. iners* (*p* = 0.7876 represented as ns in the figure). **(D)** CD16high, CD62Lhigh neutrophils as a percentage of total neutrophils measured by flow cytometry. Higher frequency of this neutrophils subset in samples co-cultured with *L. crispatus* compared to samples co-cultured with *G. vaginalis* (*p* < 0.0001), in *L. crispatus* compared to samples co-cultured with *P. bivia* (*p* < 0.0001) and in samples co-cultured with *L. crispatus* compared to samples co-cultured with *A. vaginae* (*p* < 0.0001). All *p*-values < 0.0001 in **(D)** are represented as ****. No significant difference between the co-cultures with *L. crispatus* and *L. iners* (*p* = 0.9996 represented as ns in the figure). Each symbol represents a biological replicate. Statistical differences in neutrophil frequencies between experimental conditions were determined by ANOVA with a *p*-value < 0.05 considered significant. Lines represent mean and SD.

Given the important interactions between bacteria and neutrophils, we hypothesized that the mechanism underlying increased neutrophil lifespan and accumulation was mediated by the dysbiotic bacteria. To test this, we cultured whole blood from healthy individuals (*n* = 6) with bacteria associated with BV and non-BV vaginal microbiomes and assessed neutrophils *via* flow cytometry after co-culture. *G. vaginalis, P. bivia*, and *A. vaginae* were used to represent bacteria associated with BV, and *L. iners* and *L. crispatus* represented non-dysbiotic bacteria. Lipopolysaccharide (LPS) and peptidoglycan (PGN) were used as positive controls for gram-negative and gram-positive bacteria, respectively. We observed a higher frequency of neutrophils (percentage of live CD45+ leukocytes) in co-cultures with *G. vaginalis, P. bivia*, and *A. vaginae* compared to samples cultured with *L. crispatus* (*p* < 0.0001, *p* < 0.0001, and *p* = 0.0018, respectively) and *L. iners* (*p* < 0.0001, *p* = 0.0015, and *p* = 0.0457, respectively) (Figure 3A). Neutrophil frequencies in the samples cultured with *G. vaginalis, P. bivia*, and *A. vaginae* were similar to the LPS (*p* = 0.9616, *p* = 0.9987, and *p* = 0.5930, respectively) and PGN positive controls (*p* > 0.9999, *p* = 0.7947, *p* = 0.1344, respectively), and neutrophils frequencies in samples with *L. iners* and *L. crispatus* were similar to the negative control (*p* > 0.9999, *p* = 0.9824, respectively). We observed no significant difference between samples co-cultured with *L. iners* and *L. crispatus* (*p* = 0.9321).

Additionally, we assessed the impact of these specific bacteria on neutrophil lifespan by measuring non-apoptotic, functional neutrophils (active Caspase-3 low, CD16 low, Figure 3B). Consistent with our observations *in vivo*, we observed a higher percentage of non-apoptotic neutrophils in samples co-cultured with *G. vaginalis, P. bivia*, and *A. vaginae* compared to samples cultured with *L. crispatus* (*p* = 0.0021, *p* = 0.0126, and *p* = 0.0452, respectively) and *L. iners* (*p* = 0.0010, *p* = 0.0067, and *p* = 0.0255, respectively). The neutrophils from samples with *G. vaginalis, P. bivia*, and *A. vaginae* were similar to the positive controls LPS (*p* > 0.9999, *p* = 0.9995, and *p* = 0.9709, respectively) and PGN positive controls (*p* > 0.9999, *p* = 0.9999, *p* = 0.9844, respectively), while neutrophils from samples with *L. crispatus* and *L. iners* were comparable to the negative control (*p* > 0.9999, *p* > 0.9999, respectively). Additionally, there was no significant difference between samples cultured with *L. crispatus* and *L. iners* (*p* > 0.9999). Thus, supporting our observations *in vivo*, these data demonstrate that dysbiotic bacteria can directly promote neutrophil survival and may underlie the accumulation of neutrophils.

We also assessed the functional phenotypes of neutrophils. In the samples co-cultured with *G. vaginalis, P. bivia*, and *A. vaginae*, the frequency of CD62LlowCD16high neutrophils was similar to those observed for the positive controls LPS (*p* > 0.9999, *p* = 0.5031, and *p* = 0.1944, respectively) and PGN (*p* = 0.8926, *p* > 0.9999, and *p* = 0.9826, respectively). We did, however, identify an increase in CD62LlowCD16 high neutrophils when compared to neutrophils in samples co-cultured with *L. crispatus* (*p* < 0.0001, *p* < 0.0001, and *p* = 0.0004, respectively) and *L. iners* (*p* < 0.0001, *p* = 0.0059, and *p* = 0.0293, respectively) indicating neutrophils in the presence of BV associated bacteria are more prone to suppressive functionality (Figure 3C). The *L. crispatus* and *L. iners* were similar to negative controls (*p* > 0.9999 and *p* = 0.8099, respectively), and we observed no significant difference between *L. crispatus* and *L. iners* (*p* = 0.7876).

We also observed more CD62LhighCD16high, non-activated neutrophils in our samples co-cultured with *L. crispatus* and *L. iners* compared to samples co-cultured with *G. vaginalis* (*p* < 0.0001 and *p* < 0.0001, respectively), *P. bivia* (*p* < 0.0001 and *p* < 0.0001, respectively), and *A. vaginae* (*p* < 0.0001 and *p* < 0.0001, respectively) (Figure 3D). Similarly, neutrophils from co-cultures with *G. vaginalis, P. bivia*, and *A. vaginae* were similar to the positive controls LPS (*p* = 0.8281, *p* = 0.8182, and *p* = 0.6745, respectively) and PGN (*p* = 0.8934, *p* = 0.7356, and *p* = 0.5782, respectively) while *L.crispatus* and *L. iners* samples resembled the negative controls (*p* = 0.9942 and *p* > 0.9999, respectively). We observed no difference in the non-activated neutrophils in samples co-cultured with *L. crispatus* and *L. iners* (*p* = 0.9996). Collectively, these data demonstrate that dysbiotic bacteria can directly induce the same phenotype of neutrophils observed in women with BV.

### Bacteria Associated With Vaginal Dysbiosis Impact Epithelial Barrier Integrity

**Figure 4:**
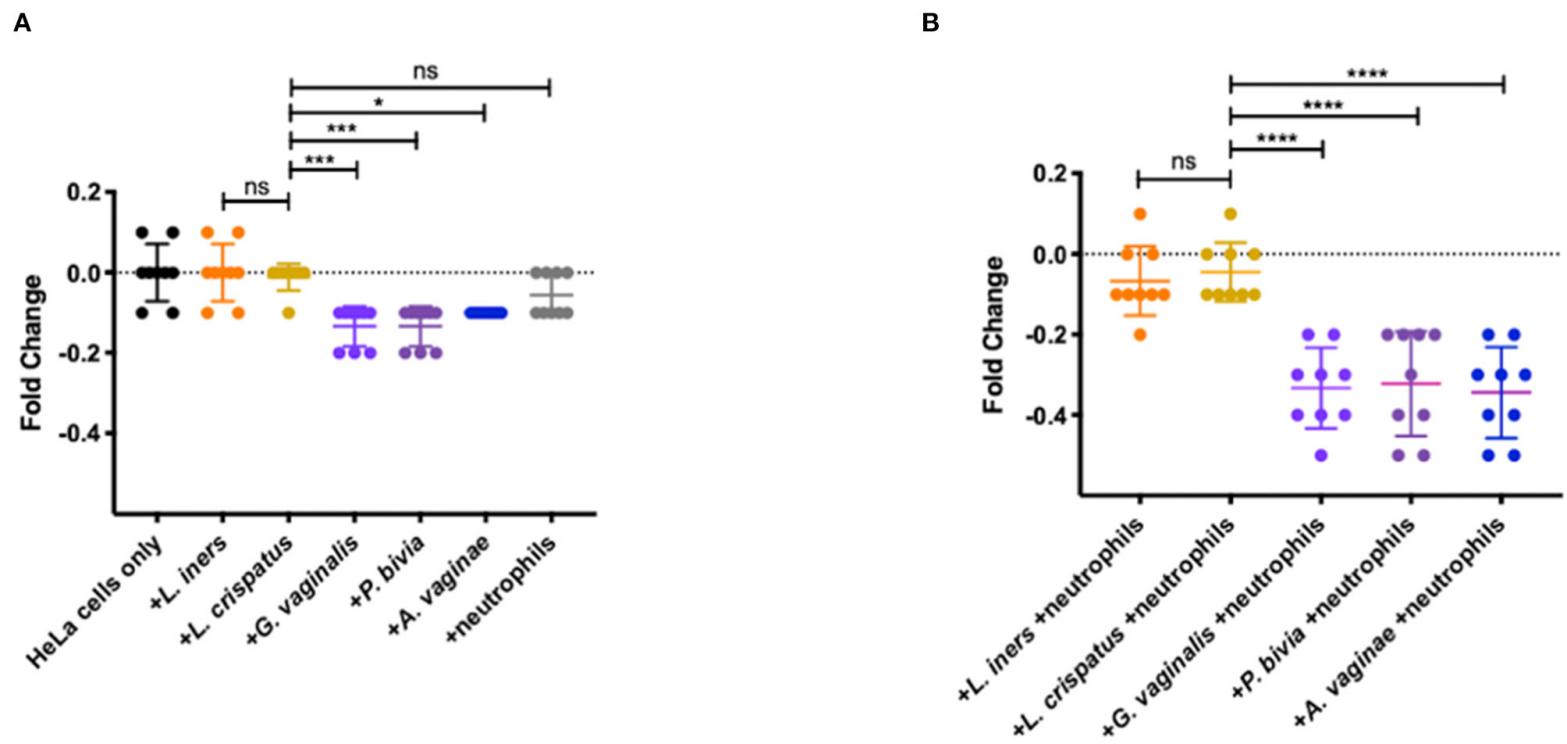
Effects of vaginal bacteria and neutrophils on HeLa cells using a TEER breakdown assay. **(A)** Fold change from baseline of TEER breakdown assay in the presence of neutrophils or vaginal bacteria. No significant differences in TEER values in wells containing neutrophils and wells containing *L. crispatus* (*p* = 0.2773, represented as ns in figure) nor in wells containing *L. iners* and *L. crispatus* (*p* = 0. 9993, represented as ns in figure). Significant decrease in fold change in wells containing the BV associated bacteria: *G. vaginalis, P. bivia*, and *A. vaginae* when compared to wells containing *L. crispatus* (*p* = 0.0001, *p* = 0.0001, and *p* = 0.0104, respectively, represented as ****, ***, and * in figure). **(B)** Fold change from baseline of TEER breakdown assay in the presence of neutrophils and vaginal bacteria. No significant difference between wells containing neutrophils and two different *Lactobacillus* species (*p* = 0.9904, represented as ns in figure). Significant decrease in fold change in TEER value in wells containing neutrophils and *G. vaginalis* or *P. bivia* or *A. vaginae* compared to co-cultures containing neutrophils and *L. crispatus* (*p* < 0.0001, *p* < 0.0001, and *p* < 0.0001, all represented in figure as ****). Statistical differences in TEER values between experimental conditions were determined by ANOVA with a *p*-value < 0.05 considered significant. Lines represent mean and SD.

To determine if activated neutrophils with reduced homeostatic apoptosis can damage epithelial barrier integrity, we isolated neutrophils from whole blood from healthy individuals. We co-cultured them with BV-associated bacteria and non-BV-associated bacteria, as described above. Transepithelial electrical resistance (TEER) is a widely used method to functionally analyze tight junctions within the physiological barriers of cell culture models and measures electrical resistance across a cellular monolayer. Combined with Lucifer Yellow transport assay, tight junction formation was verified and allowed us to assess barrier function in the presence of these neutrophil/bacteria co-cultures. Following incubation, we saw a significant decrease in fold change in wells containing the BV associated bacteria: *G. vaginalis, P. bivia*, and *A. vaginae* when compared with wells just containing *L. iners* (*p* < 0.0001, *p* < 0.0001, and *p* = 0.0026, respectively) and *L. crispatus* (*p* = 0.0001, *p* = 0.0001, and *p* = 0.0104, respectively) (Figure 4A). Differences in changes in TEER values were negligible between wells containing neutrophils only and wells with just *L. crispatus* or *L. iners* (*p* = 0.2773 and *p* = 0.5439, respectively) (Figure 4A). We saw no statistically significant difference when comparing the different *Lactobacillus* species (*p* = 0.9993). We observed a significant decrease in fold change in TEER value in wells containing neutrophils and *G. vaginalis, P. bivia*, and *A. vaginae* compared to co-cultures containing neutrophils and *L. iners* (*p* < 0.0001, *p* < 0.0001, and *p* < 0.0001, respectively) and *L. crispatus* (*p* < 0.0001, *p* < 0.0001, and *p* < 0.0001 respectively) (Figure 4B). We observed no significant difference between wells containing neutrophils and two different *Lactobacillus* species (*p* = 0.9904).

## Discussion

This study provides a detailed characterization of the impact of the vaginal microbiome on neutrophils in the female reproductive tract and the potential role of neutrophils in barrier damage. Using *ex-vivo* cytobrushes and vaginal swabs, we confirmed that neutrophil phenotype is altered in women with dysbiosis and the mechanisms by which this occurs. We found that increased neutrophil frequency is associated with decreased apoptosis and altered functionality. Furthermore, we identified *via* co-culture systems that the mechanism underlying the alterations in neutrophils is microbial dysbiosis, as we show that dysbiotic bacteria can directly induce equivalent alterations in neutrophils.

Neutrophils are important for pathogen protection, however recent studies have summarized the importance of balance within neutrophil function (24–30). For example, studies have linked increased neutrophil proteases with inflammatory cytokines and with barrier function and integrity (23, 31–34). Furthermore, studies have even identified specific immune signatures from vaginal epithelial cells elicited from specific BV-associated bacteria species, including *Prevotella bivia* and *Atopobium vaginae* (45–47). Our data allude to specific host responses to pathogenic bacteria, such as neutrophil accumulation, as a mechanism for increased HIV susceptibility during BV. This study expands on previous work identifying CD16 downregulation consistent with prolonged neutrophil survival and activity within the vagina of women with vaginitis (48). When uncontrolled, neutrophils may cause tissue damage through the release of reactive oxygen species and neutrophil proteases, both of which may damage the mucosal barrier and contribute to increased susceptibility to HIV and other STIs.

Our study also highlights the impact that vaginal dysbiosis can have on neutrophil lifespan and accumulation. Maintaining the sensitive balance of neutrophils requires controlled neutrophil clearance. Typically, neutrophils have a relatively short half-life ranging from 8 h to 5 days (49). To prevent unintended tissue damage, neutrophils undergo apoptosis to control their accumulation (50). Caspase-3 mediates the final steps of apoptosis (50), and the decreased frequency of neutrophils expressing active caspase-3 in the tissue in women with nLD vaginal microbiota highlights the impact vaginal dysbiosis can have on neutrophil lifespan. Additionally, we observed increases in total neutrophils in *in vitro* cultures with *G. vaginalis* and in cell population isolated in cytobrushes collected from women with vaginal dysbiosis. Therefore, these data and the supporting *in vitro* evidences demonstrate that pathogenic bacteria are one of the mechanism underlying neutrophil accumulation in the FGT.

Recent evidence has shown that neutrophils can act as myeloid-derived suppressor cells (MDSCs) with the ability to suppress the adaptive immune response (51). These suppressive neutrophils are associated with T cell exhaustion (52) and may contribute to diseases such as HIV infection, and may be important in promoting the latent HIV-1 reservoir (53, 54). MDSCs have shown decreased T cell function (55) and that MDSC expansion contributes to immune suppression through cytokine and cellular responses (56). Here, we confirmed that nLD communities are associated with an increase frequency of neutrophils with a suppressive phenotype using primary cervicovaginal cytobrushes. Our *in vitro* experiments demonstrated that specific taxa, such as *G. vaginalis, P. bivia*, and *A. vaginae*, could also drive this phenotype, and our 16S analysis and *in vitro* experiments indicated that healthy commensal *Lactobacillus* spp. are less likely to induce a suppressive phenotype. Of note, we did not evaluate the effect of heat killed bacteria, but we suspect that these experiments would behave similar to that of our positive controls (LPS/PGN). Together, these data provide evidence that BV-associated bacteria may be contributing to an altered neutrophil response that may weaken the local immune system, further contributing to increased susceptibility to infection.

Of note, the similar neutrophil phenotypes observed in response to culture with *L. iners* and *L. crispatus* were particularly interesting given that *L. iners* has been previously demonstrated to be more inflammatory (11), and mechanisms which underlie the differences *in vivo* that we did not observe here with neutrophils should be investigated. Maintaining the balance between anti-microbial function while preventing uncontrolled inflammation is critical, particularly in the context of HIV transmission in women with BV. These data allude to the activation of neutrophils and the favoring of a suppressive phenotype as a potential mechanism for how BV-associated bacteria increase HIV and STI transmissions through the role of neutrophils within the FRT. Further studies are needed to elucidate the exact mechanisms by which these BV associated bacteria delay apoptosis and induce phenotypic changes in neutrophils.

Importantly, this study is the first to directly demonstrate the ability of specific vaginal bacteria to reduce epithelial barrier integrity through neutrophils. Epithelial cells form dense layers with tight cell to cell junctions when cultured *in-vitro* (57). Using our TEER system, we identified decreased barrier function in the presence of neutrophils and *G. vaginalis, P. bivia*, and *A. vaginae*. Typically, pathogens invade and traverse the mucosal epithelium resulting in damaging intercellular junctions. The decrease observed in wells with these BV associated bacteria provides evidence for specific pathogenic vaginal bacteria contributing to the invasion of the mucosal epithelium. While the combination of neutrophils and BV associated bacteria induce the most significant damage to the barrier (Figure 4B), it is noteworthy that the presence of BV bacteria alone does induce mild changes in TEER values (Figure 4A). Due to this, we cannot estimate the contribution of bacteria themselves and neutrophils in inducing the additional damage observed when co-cultured together vs. when cultured individually. Future studies to dissect the cause of the increased damage could include measuring neutrophil protease activity in the presence and absence of BV associated bacteria and whether these neutrophil proteases alone could induce TEER changes. A previous study from our lab similarly identified *G. vaginalis*' impact on wounds healing and how toxins produced from *G. vaginalis* significantly reduce wound healing after 24 h (58). Our study complements the observation that neutrophil proteases cause damage (23), and these data, taken together, show that BV increases HIV and STI susceptibility through promoting damage to the tight epithelial barrier.

Lastly, our study provides additional evidence for the need to improve diagnostic tools for BV. Among our 22 samples, we identified four women diagnosed with BV by Nugent score yet had *Lactobacillus* dominant communities by 16S sequencing. Similarly, we identified four women that were not diagnosed with BV by Nugent score but had highly diverse, non-*Lactobacillus* dominant communities that represent molecular BV and contribute to increased STI and HIV infections. Further studies are needed to elucidate the observed difference between current BV diagnosis methods and 16S rRNA sequencing and how these differ in resulting BV symptoms, inflammation, and HIV/STI risk (59).

Limitations of this study include the use of *ex vivo* samples from women that were not screened for STIs. STIs are known to cause increased inflammation (60) and could contribute to alterations in neutrophil phenotypes. Furthermore, the clinical study only gathered Nugent positive or negative information and did not differentiate between BV-intermediate (Nugent score 4–6) and BV-positive (Nugent score 7–10); The reason we used Nugent score was exclusively to collect swabs from women with or without BV without identifying women in state of transition, according to the clinical protocol, and then we utilized our 16S rRNA sequencing to better characterize the vaginal microbiome to define molecular BV. We acknowledge that the Nugent score has different function, including the one to identify women in state of transition (Nugent score intermediate) that we cannot account for in this study but that should be taken in consideration in the future. However, previous studies have demonstrated that molecular BV defined using molecular sequencing is a better indication of BV status than Nugent score via clinical observation (i.e., “Nugent-BV”) (12, 61). Additionally, we focused on representative bacteria in our *in vitro* experiments, and further studies investigating the effect of other bacteria associated with BV or clinically derived bacterial stocks, and potentially mixed microbial populations, on neutrophils and barrier damage would further increase our understanding of how the complex vaginal microbiome and neutrophils together contribute to mucosal barrier damage and transmission risk. Future studies may also include cytokine/chemokine responses or transcriptome in the vaginal cells or fluid to tie together functional signaling responses with the neutrophils. Finally, understanding the importance of direct neutrophil response to bacteria vs. the response of neutrophils in the milieu of other leukocytes would further confirm these results as these experiments were conducted in whole blood. These additional studies would further elucidate the mechanisms by which BV alters female health.

Overall, this study provides an increased understanding of relationships between neutrophils, vaginal dysbiosis, and the mucosal barrier and elucidates potential mechanisms of increased HIV and STI susceptibility in women with BV. Women's health is vastly understudied and understanding the role of BV in HIV and STI acquisition is critical.

## Data Availability Statement

The data presented in the study are deposited in the NIH SRA repository at https://www.ncbi.nlm.nih.gov/bioproject accession number PRJNA719146.

## Ethics Statement

The studies involving human participants were reviewed and approved by Institutional Review Boards at St. Michael's Hospital (Toronto) and the University of Toronto. The patients/participants provided their written informed consent to participate in this study.

## Author Contributions

RC and CM designed and performed all *in vitro* experiments. RC, TH-M, and NK analyzed all flow data. AM, MY, and RK collected all *ex-vivo* cytobrushes and performed flow cytometry. CB and CD performed all 16S rRNA sequencing experiments and analyzed the data. RC, TH-M, RK, LS, RKR, and NK edited and wrote the manuscript. RK led all clinical studies. NK designed and led the overall study. All authors contributed to the article and approved the submitted version.

## Funding

These studies were supported by RO1DK112254 to NK, R01DE026327 to RKR and NK, and CIHR TMI-138656 to RK.

## Conflict of Interest

The authors declare that the research was conducted in the absence of any commercial or financial relationships that could be construed as a potential conflict of interest.

## Publisher's Note

All claims expressed in this article are solely those of the authors and do not necessarily represent those of their affiliated organizations, or those of the publisher, the editors and the reviewers. Any product that may be evaluated in this article, or claim that may be made by its manufacturer, is not guaranteed or endorsed by the publisher.

## References

[1] Borgdorff H Tsivtsivadze E Verhelst R Marzorati M Jurriaans S Ndayisaba GF. Lactobacillus-dominated cervicovaginal microbiota associated with reduced HIV/STI prevalence and genital HIV viral load in African women. ISME J. (2014) 8:1781. 10.1038/ismej.2014.26 24599071 PMC4139719

[2] Hillier SL Nugent RP Eschenbach DA Krohn MA Gibbs RS Martin DH. Association between bacterial vaginosis and preterm delivery of a low-birth-weight infant. N Engl J Med. (1995) 333:1737–42. 10.1056/NEJM199512283332604 7491137

[3] Gravett MG Hummel D Eschenbach DA Holmes KK. Preterm labor associated with subclinical amniotic fluid infection and with bacterial vaginosis. Obstet Gynecol. (1986) 67:229–37. 10.1097/00006250-198602000-00013 3003634

[4] Leitich H Bodner-Adler B Brunbauer M Kaider A Egarter C Husslein P. Bacterial vaginosis as a risk factor for preterm delivery: a meta-analysis. Am J Obstet Gynecol. (2003) 189:139–47. 10.1067/mob.2003.339 12861153

[5] Morris M Rogers P Kinghorn G. Is bacterial vaginosis a sexually transmitted infection? Sex Transm Infect. (2001) 77:63–8. 10.1136/sti.77.1.63 11158694 PMC1758329

[6] Allsworth JE Peipert JF. Severity of bacterial vaginosis and the risk of sexually transmitted infection. Am J Obstetr Gynecol. (2011) 205:113. e1–e6. 10.1016/j.ajog.2011.02.060 21514555 PMC3156883

[7] amfAR Statistics: Women and HIV/AIDS. New York, NY: Foundation for AIDS Research (2017).

[8] Schellenberg JJ Card CM Ball TB Mungai JN Irungu E Kimani J. Bacterial vaginosis, HIV serostatus and T-cell subset distribution in a cohort of East African commercial sex workers: retrospective analysis. Aids. (2012) 26:387–93. 10.1097/QAD.0b013e32834ed7f0 22095193

[9] Atashili J Poole C Ndumbe PM Adimora AA Smith JS. Bacterial vaginosis and HIV acquisition: a meta-analysis of published studies. AIDS. (2008) 22:1493. 10.1097/QAD.0b013e3283021a37 18614873 PMC2788489

[10] Ma B Forney LJ Ravel J. Vaginal microbiome: rethinking health and disease. Annu Rev Microbiol. (2012) 66:371–89. 10.1146/annurev-micro-092611-150157 22746335 PMC3780402

[11] Mclean NW Rosenstein IJ. Characterisation and selection of a Lactobacillus species to re-colonise the vagina of women with recurrent bacterial vaginosis. J Med Microbiol. (2000) 49:543–52. 10.1099/0022-1317-49-6-543 10847208

[12] McKinnon LR Achilles SL Bradshaw CS Burgener A Crucitti T Fredricks DN. The evolving facets of bacterial vaginosis: implications for HIV transmission. AIDS Res Hum Retroviruses. (2019) 35:219–28. 10.1089/aid.2018.0304 30638028 PMC6434601

[13] Gaydos CA Beqaj S Schwebke JR Lebed J Smith B Davis TE. Clinical validation of a test for the diagnosis of vaginitis. Obstet Gynecol. (2017) 130:181. 10.1097/AOG.0000000000002090 28594779 PMC5635603

[14] McClelland RS Lingappa JR Srinivasan S Kinuthia J John-Stewart GC Jaoko W. Evaluation of the association between the concentrations of key vaginal bacteria and the increased risk of HIV acquisition in African women from five cohorts: a nested case-control study. Lancet Infect Dis. (2018) 18:554–64. 10.1016/S1473-3099(18)30058-6 29396006 PMC6445552

[15] Ness RB Kip KE Hillier SL Soper DE Stamm CA Sweet RL. A cluster analysis of bacterial vaginosis–associated microflora and pelvic inflammatory disease. Am J Epidemiol. (2005) 162:585–90. 10.1093/aje/kwi243 16093289

[16] Bradshaw CS Morton AN Garland SM Morris MB Moss LM Fairley CK. Higher-risk behavioral practices associated with bacterial vaginosis compared with vaginal candidiasis. Obstetr Gynecol. (2005) 106:105–14. 10.1097/01.AOG.0000163247.78533.7b 15994624

[17] Yen S Shafer M-A Moncada J Campbell CJ Flinn SD Boyer CB. Bacterial vaginosis in sexually experienced and non–sexually experienced young women entering the military. Obstetr Gynecol. (2003) 102:927–33. 10.1016/S0029-7844(03)00858-5 14672465

[18] Moodley P Connolly C Sturm AW. Interrelationships among human immunodeficiency virus type 1 infection, bacterial vaginosis, trichomoniasis, and the presence of yeasts. J Infect Dis. (2002) 185:69–73. 10.1086/338027 11756983

[19] Cohen CR Duerr A Pruithithada N Rugpao S Hillier S Garcia P. Bacterial vaginosis and HIV seroprevalence among female commercial sex workers in Chiang Mai, Thailand. AIDS. (1995) 9:1093–7. 10.1097/00002030-199509000-00017 8527084

[20] Alcaide ML Chisembele M Malupande E Arheart K Fischl M Jones DL. cross-sectional study of bacterial vaginosis, intravaginal practices and HIV genital shedding; implications for HIV transmission and women's health. BMJ Open. (2015) 5:e009036. 10.1136/bmjopen-2015-009036 26553833 PMC4654361

[21] Kenyon C Colebunders R Crucitti T. The global epidemiology of bacterial vaginosis: a systematic review. Am J Obstet Gynecol. (2013) 209:505–23. 10.1016/j.ajog.2013.05.006 23659989

[22] Mirmonsef P Krass L Landay A Spear G. The role of bacterial vaginosis and trichomonas in HIV transmission across the female genital tract. Current HIV Res. (2012) 10:202–10. 10.2174/157016212800618165 22384839 PMC3788616

[23] Arnold KB Burgener A Birse K Romas L Dunphy LJ Shahabi K. Increased levels of inflammatory cytokines in the female reproductive tract are associated with altered expression of proteases, mucosal barrier proteins, and an influx of HIV-susceptible target cells. Mucosal Immunol. (2016) 9:194. 10.1038/mi.2015.51 26104913

[24] Hensley-McBain T NKlatt NR. The dual role of neutrophils in HIV infection. Current HIV/AIDS Rep. (2018) 15:1–10. 10.1007/s11904-018-0370-7 29516266 PMC6086572

[25] Deeks SG Lewin SR Havlir DV. The end of AIDS: HIV infection as a chronic disease. Lancet. (2013) 382:1525–33. 10.1016/S0140-6736(13)61809-7 24152939 PMC4058441

[26] Burgener A McGowan I Klatt NR HIV. and mucosal barrier interactions: consequences for transmission and pathogenesis. Curr Opin Immunol. (2015) 36:22–30. 10.1016/j.coi.2015.06.004 26151777

[27] Somsouk M Estes JD Deleage C Dunham RM Albright R Inadomi JM. Gut epithelial barrier and systemic inflammation during chronic HIV infection. AIDS. (2015) 29:43. 10.1097/QAD.0000000000000511 25387317 PMC4444362

[28] Natsui M Kawasakio K Takizawa H Hayashi SI Matsuda Y Sugimura K. Selective depletion of neutrophils by a monoclonal antibody, RP-3, suppresses dextran sulphate sodium-induced colitis in rats. J Gastroenterol Hepatol. (1997) 12:801–8. 10.1111/j.1440-1746.1997.tb00375.x 9504889

[29] Nash S Stafford J Madara JL. Effects of polymorphonuclear leukocyte transmigration on the barrier function of cultured intestinal epithelial monolayers. J Clin Invest. (1987) 80:1104–13. 10.1172/JCI113167 3116044 PMC442353

[30] Nusrat A Parkos CA Liang TW Carnes DK Madara JL. Neutrophil migration across model intestinal epithelia: monolayer disruption and subsequent events in epithelial repair. Gastroenterology. (1997) 113:1489–500. 10.1053/gast.1997.v113.pm9352851 9352851

[31] Janoff A. Neutrophil proteases in inflammation. Annu Rev Med. (1972) 23:177–90. 10.1146/annurev.me.23.020172.001141 4345523

[32] Pham CT. Neutrophil serine proteases: specific regulators of inflammation. Nat Rev Immunol. (2006) 6:541. 10.1038/nri1841 16799473

[33] Smith JA. Neutrophils, host defense, and inflammation: a double-edged sword. J Leukoc Biol. (1994) 56:672–86. 10.1002/jlb.56.6.672 7996043

[34] Pillai S Oresajo C Hayward J. Ultraviolet radiation and skin aging: roles of reactive oxygen species, inflammation and protease activation, and strategies for prevention of inflammation-induced matrix degradation–a review. Int J Cosmet Sci. (2005) 27:17–34. 10.1111/j.1467-2494.2004.00241.x 18492178

[35] Cauci S Guaschino S de Aloysio D Driussi S De Santo D Penacchioni P. Interrelationships of interleukin-8 with interleukin-1β and neutrophils in vaginal fluid of healthy and bacterial vaginosis positive women. Mol Hum Reprod. (2003) 9:53–8. 10.1093/molehr/gag003 12529421

[36] Filler SG Pfunder AS Spellberg BJ Spellberg JP Edwards J. *Candida albicans* stimulates cytokine production and leukocyte adhesion molecule expression by endothelial cells. Infect Immun. (1996) 64:2609–17. 10.1128/iai.64.7.2609-2617.1996 8698486 PMC174117

[37] Wennerholm U-B Holm B Mattsby-Baltzer I Nielsen T Platz-Christensen J Sundell G. Interleukin-1α, interleukin-6 and interleukin-8 in cervico/vaginal secretion for screening of preterm birth in twin gestation. Acta Obstet Gynecol Scand. (1998) 77:508–14. 10.1034/j.1600-0412.1998.770507.x 9654172

[38] Shaio M-F Lin P-R Liu J-Y Yang KD. Generation of interleukin-8 from human monocytes in response to *Trichomonas vaginalis* stimulation. Infect Immun. (1995) 63:3864–70. 10.1128/iai.63.10.3864-3870.1995 7558293 PMC173544

[39] Sobel JD. Vulvovaginal candidosis. Lancet. (2007) 369:1961–71. 10.1016/S0140-6736(07)60917-9 17560449

[40] Giraldo PC de Carvalho JBJ do Amaral RLG da Silveira Gonçalves AK Eleutério J Jr Guimarães F. Identification of immune cells by flow cytometry in vaginal lavages from women with vulvovaginitis and normal microflora. Am J Reprod Immunol. (2012) 67:198–205. 10.1111/j.1600-0897.2011.01093.x 22151521

[41] Hensley-McBain T Berard AR Manuzak JA Miller CJ Zevin AS Polacino P. Intestinal damage precedes mucosal immune dysfunction in SIV infection. Mucosal Immunol. (2018) 11:1429–40. 10.1038/s41385-018-0032-5 29907866 PMC6162106

[42] Caporaso JG Lauber CL Walters WA Berg-Lyons D Huntley J Fierer N. Ultra-high-throughput microbial community analysis on the Illumina HiSeq and MiSeq platforms. ISME J. (2012) 6:1621–4. 10.1038/ismej.2012.8 22402401 PMC3400413

[43] McMurdie PJ Holmes S. phyloseq: an R package for reproducible interactive analysis and graphics of microbiome census data. PLoS ONE. (2013) 8:e61217. 10.1371/journal.pone.0061217 23630581 PMC3632530

[44] Pillay J Kamp VM van Hoffen E Visser T Tak T Lammers J-W. A subset of neutrophils in human systemic inflammation inhibits T cell responses through Mac-1. J Clin Invest. (2012) 122:327–36. 10.1172/JCI57990 22156198 PMC3248287

[45] Doerflinger SY. Throop AL Herbst-Kralovetz MM. Bacteria in the vaginal microbiome alter the innate immune response and barrier properties of the human vaginal epithelia in a species-specific manner. J Infect Dis. (2014) 209:1989–99. 10.1093/infdis/jiu004 24403560

[46] Masson L Mlisana K Little F Werner L Mkhize NN Ronacher K. Defining genital tract cytokine signatures of sexually transmitted infections and bacterial vaginosis in women at high risk of HIV infection: a cross-sectional study. Sex Transm Infect. (2014) 90:580–7. 10.1136/sextrans-2014-051601 25107710

[47] Kaushic C. HIV-1 infection in the female reproductive tract: role of interactions between HIV-1 and genital epithelial cells. Am J Reprod Immunol. (2011) 65:253–60. 10.1111/j.1600-0897.2010.00965.x 21223427

[48] Beghini J Giraldo PC Riboldi R Amaral RL Eleuterio J Jr Witkin SS. Altered CD16 expression on vaginal neutrophils from women with vaginitis. Eur J Obstetr Gynecol Reprod Biol. (2013) 167:96–9. 10.1016/j.ejogrb.2012.11.008 23260596

[49] Summers C Rankin SM Condliffe AM Singh N Peters AM Chilvers ER. Neutrophil kinetics in health and disease. Trends Immunol. (2010) 31:318–24. 10.1016/j.it.2010.05.006 20620114 PMC2930213

[50] Bratton DL Henson PM. Neutrophil clearance: when the party is over, clean-up begins. Trends Immunol. (2011) 32:350–7. 10.1016/j.it.2011.04.009 21782511 PMC3151332

[51] Leliefeld PHC Wessels CM Leenen LPH Koenderman L Pillay J. The role of neutrophils in immune dysfunction during severe inflammation. Critical Care. (2016) 20:73. 10.1186/s13054-016-1250-4 27005275 PMC4804478

[52] Bowers NL Helton ES Huijbregts RPH Goepfert PA Heath SL Hel Z. Immune suppression by neutrophils in HIV-1 infection: role of PD-L1/PD-1 pathway. PLoS Pathog. (2014) 10:e1003993. 10.1371/journal.ppat.1003993 24626392 PMC3953441

[53] Cloke T Munder M Taylor G Muller I Kropf P. Characterization of a novel population of low-density granulocytes associated with disease severity in HIV-1 infection. PLoS ONE. (2012) 7:e48939. 10.1371/journal.pone.0048939 23152825 PMC3496742

[54] Shan L Deng K Shroff NS Durand CM Rabi SA Yang HC. Stimulation of HIV-1-specific cytolytic T lymphocytes facilitates elimination of latent viral reservoir after virus reactivation. Immunity. (2012) 36:491–501. 10.1016/j.immuni.2012.01.014 22406268 PMC3501645

[55] Qin A Cai W Pan T Wu K Yang Q Wang N. Expansion of monocytic myeloid-derived suppressor cells dampens T cell function in HIV-1-seropositive individuals. J Virol. (2013) 87:1477–90. 10.1128/JVI.01759-12 23152536 PMC3554138

[56] Garg A Spector SA. HIV type 1 gp120–induced expansion of myeloid derived suppressor cells is dependent on interleukin 6 and suppresses immunity. J Infect Dis. (2013) 209:441–51. 10.1093/infdis/jit469 23999600 PMC3883171

[57] Tsata V Velegraki A Ioannidis A Poulopoulou C Bagos P Magana M. Effects of yeast and bacterial commensals and pathogens of the female genital tract on the transepithelial electrical resistance of HeLa cells. Open Microbiol J. (2016) 10:90–6. 10.2174/1874285801610010090 27335621 PMC4899535

[58] Zevin AS Xie IY Birse K Arnold K Romas L Westmacott G. Microbiome composition and function drives wound-healing impairment in the female genital tract. PLoS Pathog. (2016) 12:e1005889. 10.1371/journal.ppat.1005889 27656899 PMC5033340

[59] McKinnon LR Izulla P Nagelkerke N Munyao J Wanjiru T Shaw SY. Risk factors for HIV acquisition in a prospective Nairobi-based female sex worker cohort. AIDS Behav. (2015) 19:2204–13. 10.1007/s10461-015-1118-7 26091706

[60] Castle PE Giuliano AR. Chapter 4: genital tract infections, cervical inflammation, and antioxidant nutrients—assessing their roles as human papillomavirus cofactors. JNCI Monogr. (2003) 2003:29–34. 10.1093/oxfordjournals.jncimonographs.a003478 12807942

[61] Cheu RK Gustin AT Lee C Schifanella L Miller CJ Ha A. Impact of vaginal microbiome communities on HIV antiretroviral-based pre-exposure prophylaxis (PrEP) drug metabolism. PLoS Pathog. (2020) 16:e1009024. 10.1371/journal.ppat.1009024 33270801 PMC7714160

